# Iron Status and Gestational Diabetes—A Meta-Analysis

**DOI:** 10.3390/nu10050621

**Published:** 2018-05-15

**Authors:** Yachana Kataria, Yanxin Wu, Peter de Hemmer Horskjær, Thomas Mandrup-Poulsen, Christina Ellervik

**Affiliations:** 1 Department of Laboratory Medicine, Boston Children’s Hospital, 300 Longwood Avenue, Boston, MA 02215, USA; Yanxin.Wu@childrens.harvard.edu (Y.W.); Christina.Ellervik@childrens.harvard.edu (C.E.); 2 Harvard Medical School, Boston, MA 02115, USA; 3 Department of Biomedical Sciences, Faculty of Health and Medical Sciences, University of Copenhagen, Nørre Alle 41, 2200 Copenhagen, Denmark; horskjaer@sund.ku.dk (P.d.H.H.); tmpo@sund.ku.dk (T.M.-P.); 4 Department of Clinical Medicine, Faculty of Health and Medical Sciences, University of Copenhagen, Nørre Alle 41, 2200 Copenhagen, Denmark

**Keywords:** iron, ferritin, transferrin, gestational diabetes, pregnancy

## Abstract

A meta-analysis of the association of iron overload with gestational diabetes mellitus (GDM) may inform the health debate. We performed a meta-analysis investigating the association of iron biomarkers and dietary iron exposure with GDM. We identified 33 eligible studies (*N* = 44,110) published in 2001–2017. The standardized mean differences (SMD) in women who had GDM compared to pregnant women without were 0.25 µg/dL (95% CI: 0.001–0.50) for iron, 1.54 ng/mL (0.56–2.53) for ferritin, 1.05% (0.02 to 2.08) for transferrin saturation, and 0.81 g/dL (0.40–1.22) for hemoglobin. Adjusted odds ratio for GDM were 1.58 (95% CI: 1.20–2.08) for ferritin, 1.30 (1.01–1.67) for hemoglobin, and 1.48 (1.29–1.69) for dietary heme intake. We did not find any differences in TIBC or transferrin concentration in women with and without GDM. We also did not find any association of increased transferrin receptor or increased intake of total dietary iron, non-heme iron or supplemental iron, with increased odds ratios for GDM. Considerable heterogeneity was present among the studies (0–99%), but no evidence of publication bias. Accumulating evidence suggests that circulating and dietary iron biomarkers among pregnant women are associated with GDM, but the results should be interpreted with caution due to the high heterogeneity of analyses. Randomized trials investigating the benefits of iron reduction in women at high risk for GDM are warranted.

## 1. Introduction

Body iron is tightly regulated and dependent on nutritional needs and availability [1]. Adequate iron is critical for β**-**cell function and glucose homeostasis but excess iron beyond the need increases systemic oxidative stress [2]. Iron deficiency is common among pregnant women and remains a global public health concern. The World Health Organization (WHO) recommends intake of 30–60 mg of elemental iron during pregnancy to prevent maternal iron deficiency anemia and to ensure adequate fetal iron stores [1]. However, the health policy about the amount of iron supplementation during pregnancy varies between countries. 

It is evident that pregnant women have an increased demand for iron due to physiological expansion of blood volume, needs of the fetus, and placental growth. Gestational diabetes mellitus (GDM) is associated with risks for the mom and the fetus. O’Sullivan et al. originally recommended the first screening and diagnostic criteria in the 1960s [3]. Since then, there has been debate about screening and diagnostic criteria for GDM. As such, various GDM diagnostic criteria have been adopted around the world by governing agencies, and the criteria have been modified over the years [3,4]. Collectively, this results in variable prevalence rates of GDM throughout the world. GDM is the most common metabolic disorder of pregnancy, and increasing incidence rates of GDM are thought to reflect the global rise in the incidence of type 2 diabetes mellitus (T2DM) related to energy intake in excess of energy expenditure [5]. Therefore, strategies addressing effective prevention, proper diagnosis and treatment are warranted. Fetal complications include obstetric complications related to macrosomia, neonatal hypoglycemia, perinatal mortality, congenital malformations, hyperbilirubinemia, polycythemia, hypocalcemia and respiratory distress syndrome [6]. The offspring are also at higher risk for glucose intolerance, diabetes and obesity in later years [6]. Maternal complications in women who develop GDM include hypertension, preeclampsia, cesarean delivery and increased risk of developing diabetes after pregnancy [5,6]. 

It has been debated whether excess endogenous and exogenous (supplemental) iron is associated with GDM, but so far the studies have been inconsistent and heterogeneous with respect to measurement of iron exposure, ascertainment of GDM status, study design, and population demographics [7,8,9,10,11,12,13,14,15,16,17,18,19,20,21,22,23,24,25,26,27,28,29,30,31,32,33,34,35,36,37,38]. There have been a few meta-analyses of observational studies published to date, but they are not comprehensive and fail to provide conclusive evidence as to whether iron supplementation during pregnancy would pose any risk to development of GDM. Two previous randomized clinical trials in pregnant women failed to find any significant difference in the incidence of GDM in the iron supplement and placebo groups but these studies suffered from limitations in trial design, compliance, and ascertainment of exposure and outcome [19,39]. Most recently, Zhang et al. published a qualitative systematic review that critically examined the association between dietary iron intake, iron status and gestational diabetes, but the study did not provide quantitative estimates across the studies. Therefore, the aim of this study was to perform a quantitative, meta-analysis of the association of dietary iron intake, iron supplementation, and circulating iron biomarkers with GDM to inform the health debate.

## 2. Methods 

### 2.1. Study Strategy

The study was conducted in accordance to Meta-analysis of Observational Studies in Epidemiology (MOOSE) recommendations.

A systematic search of studies published before 27 November 2017 was conducted utilizing a PubMed Search. The search was designed to capture records for articles describing the effect of iron intake and serum biomarkers of iron and GDM: (“iron” [MeSH Terms] OR “iron” [All Fields]) AND (“diabetes, gestational” [MeSH Terms] OR (“diabetes” [All Fields] AND “gestational” [All Fields]) OR “gestational diabetes” [All Fields] OR (“gestational” [All Fields] AND “diabetes” [All Fields])). Additional articles were captured by references and citations found in the original search and from the PubMed option “Similar Articles.” 

### 2.2. Exposure

Exposure was defined as circulatory or dietary biomarkers of iron: hemoglobin (g/dL), ferritin (ng/mL), total iron binding capacity (TIBC) (µg/dL), transferrin (mg/dL), transferrin saturation (%), and transferrin receptor (mg/L). Dietary iron biomarkers included: total dietary iron with supplements, dietary iron without supplements, non-heme iron, heme iron, and supplemental iron. 

### 2.3. Outcomes

A diagnosis of gestational diabetes status was based either on oral glucose tolerance test (OGTT), medical record, and/or self-report. More specifically, the OGTT diagnosis cutoff values utilized depend on which criteria were used by the study (i.e., WHO, ADA, etc.). Specific criteria for each study are specified in Table 1. 

### 2.4. Inclusion Criteria

Studies were selected if they reported an association of iron biomarkers and GDM. 

The following criteria were required for eligibility: (A) mean, standard deviation (SD), and N of the continuous exposures in pregnant women with vs. without GDM, or (B) unadjusted and/or adjusted odds ratio and 95% CI for the binary outcomes in pregnant women with vs. without GDM, or (C) raw N for the 2 × 2 tables to calculate the odds ratio and 95% CI for the binary outcomes in pregnant women with vs. without GDM.

### 2.5. Exclusion Criteria

We excluded abstracts, reviews, commentaries, studies with inadequate data, and studies in languages other than English. “Inadequate data” is defined as data that were presented such that it was impossible to extract or calculate the necessary values for inclusion into a meta-analysis. 

### 2.6. Study Selection Process

**Figure 1:**
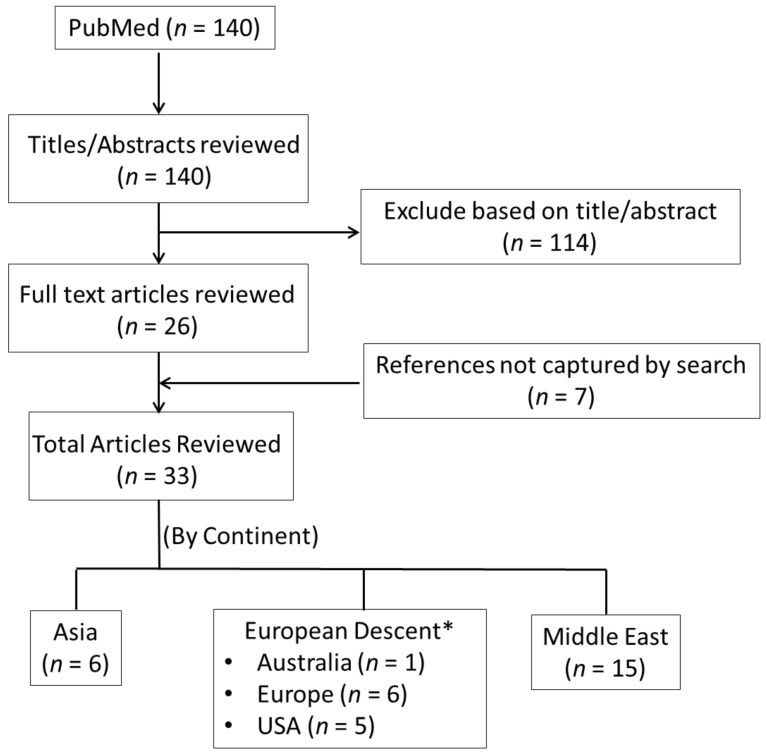
Selection of studies. Note: Australia, Europe, and USA were clustered due to the limited number of studies in individual analyses.

One author (YK) first examined the study titles and abstracts. All studies identified as potentially relevant to the topic were eligible for a full-text review. The study selection process is shown in Figure 1. A list of selected studies can be found in Table 1. A total of 33 eligible studies were identified [7,8,9,10,11,12,13,14,15,16,17,18,19,20,21,22,23,24,25,26,27,28,29,30,31,32,33,34,35,36,37,38].

### 2.7. Risk of Bias and Study Quality Assessment

The quality of each study was evaluated and scored using the nine-star Newcastle–Ottawa Scale (NOS), a tool used for quality assessment of nonrandomized studies [40]. This was done independently by two authors (YK & YW). Studies were evaluated based on selection, comparability, exposure, and outcome, and scored with a maximum of nine points. Scores above five indicate moderate to high study quality. The NOS for cohort and case-control studies was retrieved from: http://www.ohri.ca/programs/clinical_epidemiology/nosgen.pdf.

### 2.8. Data Extraction 

Two authors (YK & YW) extracted information from the selected studies. The following variables were obtained from each study: author, size of study, year of publication, journal, name of study, assessment of GDM, GDM diagnosis criteria, assay type, the number of people with outcome/no outcome in exposed and non-exposed groups, odds ratio with 95% confidence interval (CI) or standard error (SE), mean and SD for continuous variables for exposed and non-exposed groups, and adjustment variables. Table 1 shows details of the selected studies and the data extracted from each study. 

### 2.9. Data Synthesis and Meta-Analysis

Analysis was performed using STATA statistical software version 13.1. We combined summary estimates from all the studies in a meta-analysis. 

We used the *metan* command to calculate random effects summary estimates in STATA. Comparing dietary and serum iron status to GDM vs. no GDM, we calculated weighted mean differences (WMD) and standardized mean differences (SMD) for continuous variables, and a pooled odds ratio for blood levels of ferritin, transferrin receptor, hemoglobin, and dietary iron intake. We meta-analyzed the unadjusted and adjusted data separately. If studies presented odds ratios parsed by tertiles or quartiles of iron status, we calculated a within-study fixed effect odds ratio for binary GDM status (yes/no) before we meta-analyzed the odds ratios across studies. Statistical heterogeneity was assessed by the I^2^ statistic, which is a measure of between study variance, and the corresponding Cochran’s Q-statistic *p*-value (p(het)). To investigate the validity and robustness of the meta-analysis of the mean differences in iron, ferritin, and hemoglobin, for which we had the most studies, we explored heterogeneity in sensitivity analyses by stratifying on study size, continent, and study design, and we performed leave-one-out analyses. Publication bias was measured by Begg’s test and Egger’s test and by visually assessing funnel plots. 

## 3. Results 

After removing duplicates, 33 unique articles were identified (Figure 1). We included 33 studies in the meta-analysis on blood biomarkers of iron and dietary iron intake and its association with gestational diabetes. The Newcastle–Ottawa qualitative assessment scale of bias revealed a score of 5–8 in case-control studies and 6–9 in cohort studies (Table S1). Fifteen studies were from the Middle East, six from Europe, six from Asia, six from the USA, and one from Australia.

The studies were published from 2001 to 2017. In total, 44,110 pregnant women were included (age range 19 to 41 years). 

### 3.1. Serum Iron 

**Figure 2:**
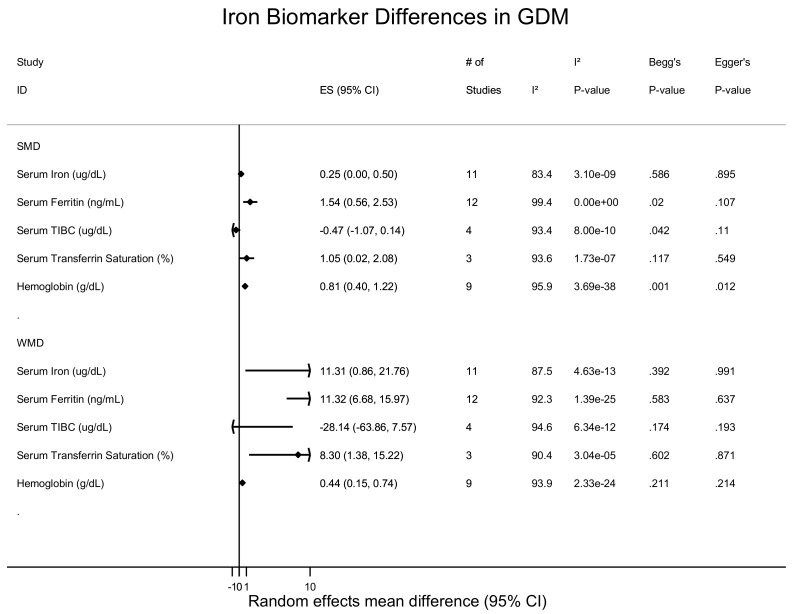
Blood and dietary iron biomarkers and gestational diabetes status (mean difference).

The SMD and WMD of serum iron in women who had GDM compared to pregnant women without were 0.25 µg/dL (95% CI: 0.001 to 0.50; I^2^ = 83.4%, p(het) = 3.10 × 10^−9^) and 11.31 µg/dL (95% CI: 0.87 to 21.75 I^2^ = 87.5%, p(het) = 4.63 × 10^−13^), respectively (Figure 2, Figures S1 and S2).

### 3.2. Serum Ferritin 

**Figure 3:**
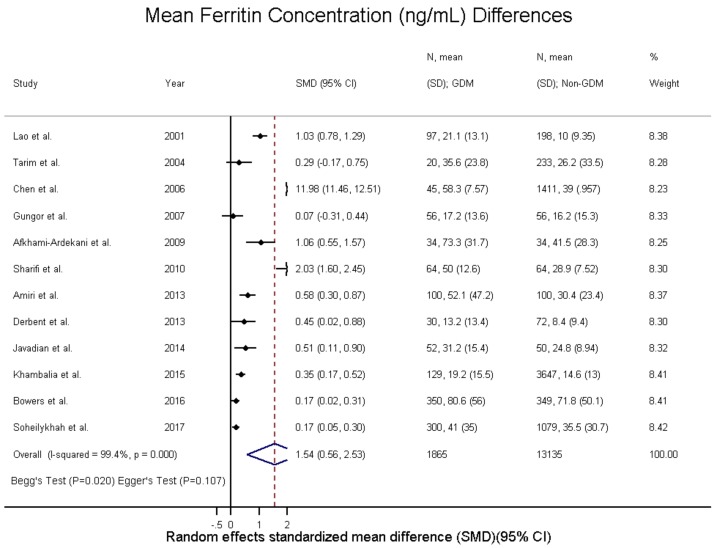
Ferritin concentration (ng/mL) differences in GDM—standardized mean differences.

**Figure 4:**
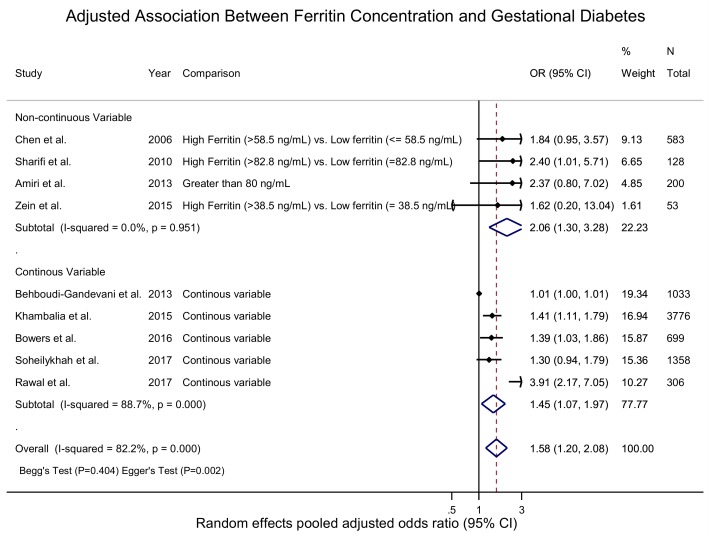
Adjusted association between ferritin concentration (ng/mL) and GDM—adjusted odds ratio.

The SMD and WMD of serum ferritin in women who had GDM compared to pregnant women without were 1.54 ng/mL (95% CI: 0.56 to 2.53; I^2^ = 99.4.0%, p(het) = 0) and 11.33 n/g/dL (95% CI: 6.68 to 15.97; I^2^ = 92.4%, p(het) = 1.39 × 10^−25^), respectively (Figure 2 and Figure 3, and Figure S3). Increased ferritin concentration was associated with GDM with an unadjusted odds ratio of 1.84 (95% CI: 1.51 to 2.23; I^2^ = 37.1%, p(het) = 1.1 × 10^−1^) (Figure 2 and Figure S4) and an adjusted odds ratio of 1.58 (95% CI: 1.20 to 2.08; I^2^ = 0.0%, p(het) = 3.83 × 10^−7^) (Figure 4 and Figure S4). 

### 3.3. Total Iron Binding Capacity

The SMD and WMD of total iron binding capacity (TIBC) in women who had GDM compared to pregnant women without were −0.47 µg/mL (95% CI: −1.07 to 0.14; I^2^ = 93.4%, p(het) = 8.0 × 10^−10^) and −28.14 µg/dL (95% CI: −63.86 to 7.57; I^2^ = 94.6%, p(het) = 1.39 × 10^−25^), respectively (Figure 2, Figures S5 and S6). 

### 3.4. Transferrin Saturation and Transferrin Receptor

The SMD and WMD of transferrin saturation in women who had GDM compared to pregnant women without were 1.05% (95% CI: 0.02 to 2.08; I^2^ = 93.6%, p(het) = 1.73 × 10^−7^) and 8.30% (95% CI: 1.38 to 15.22; I^2^ = 90.4%, p(het) = 3.04 × 10^−5^), respectively (Figure 2, Figures S7 and S8).

**Figure 5:**
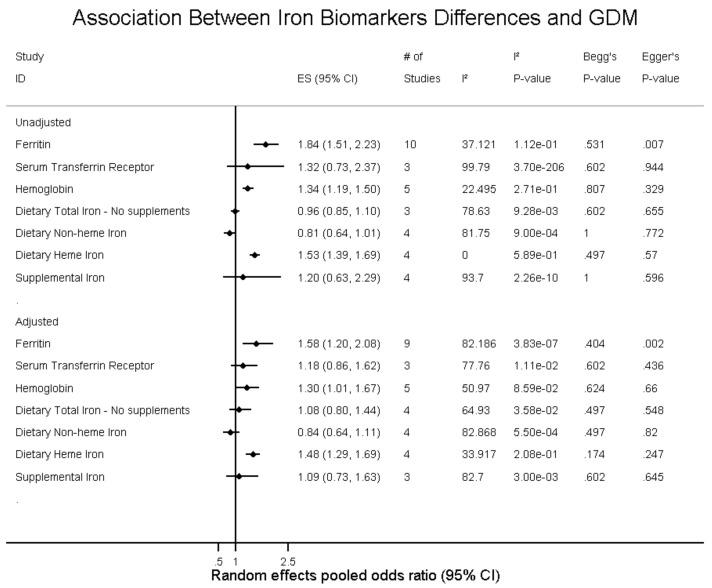
Differences in iron and gestational diabetes status (odds ratio).

Increased transferrin receptor concentrations were associated with GDM with an unadjusted odds ratio of 1.32 (95% CI: 0.73 to 2.37; I^2^ = 99.8%, p(het) = 3.70 × 10^−206^) (Figure 5 and Figure S12) and an adjusted odds ratio of 1.18 (95% CI: 0.86 to 1.62; I^2^ = 77.8%, p(het) = 1.11 × 10^−2^) (Figure 5, Figures S9 and S10). 

### 3.5. Hemoglobin

**Figure 6:**
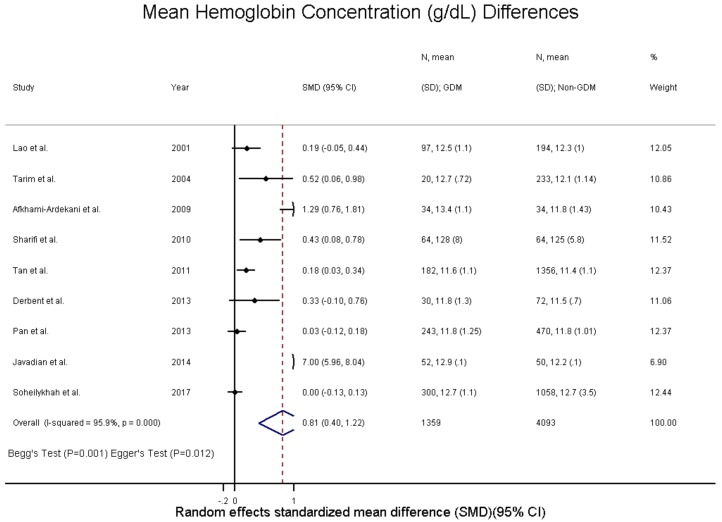
Hemoglobin concentration (g/dL) differences in GDM—standardized mean difference.

The SMD and WMD for hemoglobin levels in women who had GDM compared to pregnant women without were 0.81 g/dL (95% CI: 0.40 to 1.22; I^2^ = 95.9%, p(het) = 3.69 × 10^−38^) and 0.44 g/dL (95% CI: 0.15 to 0.74; I^2^ = 93.9%, p(het) = 2.33 × 10^−24^), respectively (Figure 2 and Figure 6 and Figure S11). Increased hemoglobin concentration was associated with GDM with an unadjusted odds ratio of 1.34 (95% CI: 1.19 to 1.50; I^2^ = 22.5%, p(het) = 2.71 × 10^−1^) (Figure 5 and Figure S12) and an adjusted odds ratio of 1.30 (95% CI: 1.01 to 1.67; I^2^ = 51.0%, p(het) = 8.59 × 10^−2^) (Figure 5 and Figure S13).

### 3.6. Dietary Iron Biomarkers

Total dietary iron, not including supplements, was not associated with GDM. The unadjusted OR was 0.96 (95% CI: 0.85 to 1.10; I^2^ = 78.6%, p(het) = 9.28 × 10^−3^) (Figure 5 and Figure S14) and the adjusted OR was 1.08 (95% CI: 0.80 to 1.44; I^2^ = 64.9%, p(het) = 3.58 × 10^−2^) (Figure 5 and Figure S15).

Dietary non-heme iron intake was not associated with GDM, with unadjusted OR of 0.80 (95% CI: 0.64 to 1.01; I^2^ = 81.8%, p(het) = 9.00 × 10^−4^) and adjusted OR of 0.84 (95% CI: 0.64 to 1.11; I^2^ = 82.9%, p(het) = 5.50 × 10^−4^) (Figure 5, Figures S16 and S17). 

Dietary heme iron intake was associated with GDM with an unadjusted OR of 1.53 (95% CI: 1.39 to 1.69; I^2^ = 0.0%, p(het) = 5.89 × 10^−1^) and adjusted OR of 1.48 (95% CI: 1.29 to 1.69; I^2^ = 33.9%, p(het) = 2.09 × 10^−1^) (Figure 5, Figures S18 and S19). 

Lastly, supplemental iron intake was not associated with GDM with an unadjusted OR of 1.20 (95% CI: 0.63 to 2.29; I^2^ = 93.7%, p(het) = 2.26 × 10^−10^) and adjusted OR of 1.09(95% CI: 0.73 to 1.63; I^2^ = 82.7%, p(het) = 3.00 × 10^−3^) (Figure 5, Figures S20 and S21).

### 3.7. Heterogeneity and Publication Bias and Sensitivity Analysis 

I^2^ heterogeneity varied from 0–99% but there was no publication bias present in the meta-analyses (Figure 2 and Figure 5). Stratifying the meta-analysis by study size, showed that larger studies had smaller mean iron differences with narrow confidence intervals compared to smaller studies, but study size did not influence the mean differences observed for ferritin and hemoglobin concentrations (Figure S22). Stratifying the meta-analysis by geographical location revealed that studies from the Middle East and Asia had the larger and more variable mean differences in iron and hemoglobin levels (Figure S23), whereas mean differences in ferritin levels were higher among women of European descent. Stratification by study design revealed a variable pattern and heterogeneity (I^2^: 0–99%) amongst all studies for mean differences in iron, ferritin, and hemoglobin levels (Figure S24). Lastly, no gross changes were found in iron, ferritin, and hemoglobin mean differences compared to pooled sensitivity analysis conducted by leave-one-out analysis (Figures S25–S27). 

## 4. Discussion

There has been growing literature suggesting that elevated iron concentration is associated with the development of GDM. Understanding the relationship is imperative for designing future randomized control trials to determine if pregnant women can benefit from early intervention. We conducted a meta-analysis to determine if circulatory and dietary iron biomarkers are associated with development of gestational diabetes. The findings from this meta-analysis suggested that mean differences in circulating iron, ferritin, hemoglobin, and transferrin saturation were higher in women with GDM compared to women without GDM, and increased ferritin, hemoglobin, and dietary heme intake were associated with increased odds ratios for GDM. We did not find any meaningful differences in TIBC and transferrin concentration in women with and without GDM. We also did not find any association of increased transferrin receptor or increased intake of total dietary iron, non-heme iron or supplemental iron, with increased odds ratio for GDM.

Ferritin concentrations were significantly and consistently associated with GDM across various observational studies and other meta-analyses [41,42,43,44,45]. Of note, the relationship persisted across different thresholds of high vs. low ferritin concentrations across different studies. It is also unclear what threshold of iron elevation is associated with GDM. Ferritin is a marker utilized to assess total body iron stores and it is also an acute phase reactant. The hormone hepcidin is the body’s main regulator of systemic iron homeostasis, and it is secreted in response to iron loading and inflammation [46]. Hepcidin has been associated with inflammatory states in humans [46,47]. The role of hepcidin in pregnancy is poorly understood, and only two studies have examined the association between GDM and hepcidin, both reporting increased hepcidin levels in GDM [15,24]. An increased maternal serum hepcidin level in GDM could potentially be due to increased body iron stores or inflammation. Interestingly, Rawal et al. found that the relationship persisted after adjusting for inflammation status (i.e., CRP levels). In a similar fashion, the association observed between ferritin and GDM could be reflective of underlying inflammation and not iron status. Moreover, markers of inflammation such as C-Reactive Protein (CRP) have also been associated with GDM [48]. To date, only a handful of studies simultaneously assessed the CRP and ferritin with GDM [10,15,38,49,50]. Chen et al. showed that individuals with higher serum ferritin and CRP levels had the greatest risk of GDM. This is consistent with Jiang et al. who has reported that high serum ferritin and CRP are independent risk factors for Type 2 diabetes [51]. Collectively this suggests that inflammation may mediate the development of GDM. However, in our analysis, other non-acute phase reactant markers such as serum iron and hemoglobin were also associated with GDM.

No single circulating iron biomarker alone is reflective of true iron status. Clinical utility of iron, ferritin, transferrin, transferrin saturation, soluble transferrin receptor are best when used in combination. However, the majority of the included studies failed to measure all iron biomarkers.

Serum iron measures the amount of circulating iron; our pooled estimates suggested that there was a positive and significant association between iron concentration and GDM. Animal model studies have confirmed that glucose tolerance is decreased by iron supplementation and is increased by iron depletion [52]. Whereas, transferrin, an iron transport protein, and transferrin saturation is readily calculated to serve as a sensitive indicator of functional iron deficiency. Our pooled estimates suggest that serum transferrin saturation is associated with GDM but not transferrin levels. Soluble transferrin receptor is increased in iron deficiency but unlike ferritin is not confounded by inflammation. All three studies published to date have found no significant association between transferrin receptor and GDM risk [13,15,38]. It is possible that no association was seen since transferrin receptor levels tend to rise only in the presence of a functional iron deficiency. Collectively, there were a limited number of studies examining these associations and thus results should be evaluated critically. 

Hemoglobin concentration is affected by hemodilution during pregnancy, and a decreased concentration defines anemia; however, increased hemoglobin may be reflective of iron overload but may also reflect heavy smoking, high altitude, or a bone marrow disorder. In the meta-analysis, elevated hemoglobin concentration was associated with GDM. 

Iron status of the body is regulated by dietary intake of the individual and also dependent on the type of dietary iron (i.e., heme vs. non-heme). Heme and non-heme iron are derived from different food sources and have different absorption mechanisms. Heme iron is derived largely from hemoglobin and myoglobin in meat, poultry, and fish. On the contrary plants, dairy products, and supplements are sources of non-heme iron. Dietary heme iron was positively and significantly associated with GDM but total dietary iron and non-heme iron were not associated with GDM. This finding could be attributable to the different absorption rates of heme and non-heme iron. Additionally the absorption of heme iron is less influenced by other dietary factors compared to non-heme iron [53]. The negative and non-significant association of non-heme with GDM could be serving as a proxy for other dietary components that are reflective of a healthier diet but these findings are inconsistent. As such, fiber and polyunsaturated fats have been reported to be inversely associated with GDM risk [54,55]. 

In support of our findings, a case-control study showed that the iron overload C282Y allele frequency is higher in women with GDM than in healthy pregnant women, suggesting a genetic susceptibility to the development of GDM [56]. Our group has also previously shown that iron overload is associated with diabetes [57,58]. Higher levels of iron contribute to more reactive oxygen species and oxidative stress, ultimately leading to damage of the pancreatic beta cells and impairing insulin synthesis [59,60]. Reduction of the iron burden in type 2 diabetes animal models improve beta cell function [2]. It is also plausible that iron excess might interfere with glucose metabolism and trigger insulin resistance rather than impairing beta cells function. Animal models have shown that iron overload results in insulin resistance and hepatic glucose production [61]. This maybe mediated through the pancreatic beta-cells as it has a poor antioxidant capacity making them more susceptible to oxidative damage ultimately leading to impaired insulin synthesis. 

Our meta-analysis is the most recent that comprehensively, critically, and quantitatively assesses the association between serum iron biomarkers and dietary biomarkers with gestational diabetes. Previous meta-analyses have either only qualitatively assessed the current data and/or are not comprehensive [13,41,42,44,45]. However, our study has limitations that should to be considered. Each meta-analysis had a variable number of studies included (3 to 12), which highlights the lack and need for additional studies assessing the association between iron status and GDM. Majority of the studies utilized either the ADA, WHO, or the Carpenter and Coustan OGTT diagnostic criteria. Assessing the studies by continent, majority of the studies (15 in total) were from the Middle East, six from Europe, six from Asia, five from the USA, and one from Australia. The GDM status was different in different regions of the world, and amount of glucose for OGTT varied between countries. These factors most likely contributed to the geographical heterogeneity that was observed in the meta-analyses. There were also studies with self-reported GDM, which could introduce misclassification bias and potentially affect the observed results. Additionally, the selection of iron biomarkers to assess iron status varied among all the selected studies, which could potentially introduce further bias in the observed results, and none of the studies used iron isotopes to measure iron absorption. Moreover, the heterogeneous selection of assays across studies may introduce bias, which may prevent us from observing a true association between iron status and GDM; however, the use of the standardized mean difference largely reduces bias due to differences between assays. Additionally, almost all of the studies were observational in nature thus residual confounding can be expected. Recall bias may also be present in the questionnaire assessments of dietary iron intake. The presence of random error also attenuates the associations observed in this study and favors the null hypothesis. 

The meta-analyses were based on observational studies, which are prone to confounding and reverse causation. Two previous randomized clinical trials (RCT) have been conducted investigating the effect of iron supplementation in healthy pregnant women and most with some degree of anemia, but did not find any association [19,39]. These studies suffered from limitations in trial design, compliance, and ascertainment of exposure and outcome. RCT studies would confirm findings seen across the literature and help determine if reducing iron status would prevent diabetes and/or improve glycemic control in patients with varying degrees of glucose intolerance; but to this date, no RCTs have been conducted in women with first-time GDM or in women with a second pregnancy with a previous history of GDM investigating the effects of iron supplementation on the mother and the fetus. These studies are needed to identify if certain high-risk groups could benefit from iron reduction or simply no iron supplement during pregnancy. Furthermore, concerns regarding iron supplementation in women with adequate levels ought to be investigated.

In conclusion, the meta-analysis suggested that mean differences in circulating iron, ferritin, hemoglobin, and transferrin saturation were higher in women with GDM compared to women without GDM, and increased ferritin, hemoglobin, and dietary heme intake were associated with increased odds ratios for GDM. Results should be interpreted with caution as the pooled estimates had high heterogeneity, potentially due to geographical location, GDM assessment, and selection of iron biomarkers and assays. The lack of association does not necessarily support the null hypothesis as some analyses included only a few studies and thus have wider confidence intervals. There is a clear need for larger and systematically conducted randomized controlled trials utilizing all the available iron biomarkers to elucidate the potential benefit and risk of iron repletion among replete pregnant women and in pregnant women at high risk of developing GDM. 

## Figures and Tables

**Table 1:** Characteristics of studies included in the meta-analyses.

Author	Year	Country	Design	N Cases	N Total	Ascertainment of GDM	Age ^a^	Dietary Heme Iron	Dietary Non-Heme Iron	Dietary Total Iron	Dietary Supplemental Iron	Serum Iron	Serum Ferritin	Serum Transferrin Receptor	Hemoglobin	CRP	Serum TIBC	Serum Transferrin Saturation	Serum Ferritin Assay
Marí-Sanchis et al.	2017	Spain	Cohort	172	3298	Self Report	28.7	X	X	X									
Behboudi-Gandevani et al.	2013	Iran	Cohort	72	1033	OGTT	27.6			X		X	X		X				NR
Bowers et al.	2011	US	Cohort	867	13,475	Self Report	31.4	X	X	X	X								
Chen et al.	2006	US	Cohort	45	1456	OGTT	22.1						X		X	X			Immunoradiometric
Darling et al.	2016	US	Cohort	316	7229	Self Report	NR	X	X										
Helin et al.	2012	Finland	Cohort	72	399	Medical Record/OGTT	29.3			X									
Khambalia et al.	2015	Australia	Cohort	129	3776	Medical Record	NR						X	X		X			ELISA
Qiu et al.	2011	US	Cohort	158	3158	Medical Record	32.7	X	X										
Rawal et al.	2017	US	Case-Control	107	321	Medical Record	30.4						X	X		X			Immunoturbidimetric
Soheilykhah et al.	2017	Iran	Cohort	300	1358	OGTT	20.3–31.4					X	X		X		X		ELISA
Soubasi et al.	2010	Greece	Cohort	6	63	Medical Record	24–37						X						ELISA
Tarim et al.	2004	Turkey	Cohort	20	253	OGTT	21.8–32.3						X		X				NR
Zein et al.	2015	Lebanon	Cohort	16	104	OGTT	20–33						X		X	X			Chemiluminescence
Chan et al.	2009	China	Randomized Control Trial	116	1164	OGTT	31.1–31.5				X								
Afkhami-Ardekani et al.	2009	Iran	Case-Control	34	68	OGTT	NR					X	X		X		X	X	Immunoradiometric
Al Saleh et al.	2004	Kuwait	Case-Control	15	30	Not Reported	23.1					X							
Al Saleh et al.	2007	Kuwait	Case-Control	10	21	Not Reported	28.0–33.7					X							
Amiri et al.	2013	Iran	Case-Control	100	200	OGTT	19.6–31.0					X	X				X		Immunoradiometric
Derbent et al.	2013	Turkey	Case-Control	30	102	OGTT	23.9–37					X	X		X				Electrochemiluminescence
Gungor et al.	2007	Turkey	Case-Control	56	112	OGTT	21.1–33.9						X						MEIA
Javadian et al.	2014	Iran	Case-Control	52	102	OGTT	22.3–37.8						X		X				Immunoradiometric
Kaygusuz et al.	2013	Turkey	Case-Control	30	58	OGTT	28.7–32.8					X					X	X	
Ozyer et al.	2014	Turkey	Case-Control	35	105	OGTT	26.8–34.1									X			
Sharifi et al.	2010	Iran	Case-Control	64	128	OGTT	25.1–34.9						X		X				Immunoradiometric
Wang et al.	2002	China	Cross-sectional	46	136	OGTT	NR					X							
Akhlaghi et al.	2012	Iran	Case-Control	30	60	OGTT	25–30					X							
Pan et al.	2013	China	Cross-sectional	243	713	OGTT	29.5								X				
Lao et al.	2001	China	Cross-sectional	97	291	OGTT	34.9–37.4					X	X		X			X	MEIA
Lao et al.	2002	China	Cross-sectional	94	730	OGTT	29.7								X				
Tan et al.	2011	Malaysia	Cohort	182	1538	OGTT	24.6–36.9								X				
Bowers et al.	2016	Denmark	Case-Control	350	699	OGTT	25.7–36.5						X	X					Immunoturbidimetric
Bo et al.	2009	Italy	Case-Control	500	1000	OGTT	28.3–41				X								
Palma et al.	2008	Spain	Case-Control	41	930	Medical Record	20–25				X								

a: Mean or ranges provided. NR = Not reported; MEIA = Microparticle enzyme immunoassay.

## Supplementary Materials

The following are available online at http://www.mdpi.com/2072-6643/10/5/621/s1. Figure S1: Iron Concentration (µg/dL) Differences in GDM—Standardized Mean Differences; Figure S2: Iron Concentration (µg/dL) Differences in GDM—Weighted Mean Difference; Figure S3: Ferritin Concentration (ng/mL) Differences in GDM—Weighted Mean Difference; Figure S4: Association Between Ferritin Concentration and GDM—Unadjusted Odds Ratio; Figure S5: TIBC Concentration (µg/dL) Differences in GDM—Standardized Mean Differences; Figure S6: TIBC Concentration (µg/dL) Differences in GDM—Weighted Mean Differences; Figure S7: Transferrin Saturation (%) Differences in GDM—Standardized Mean Differences; Figure S8: Transferrin Saturation (%) Differences in GDM—Weighted Mean Differences; Figure S9: Association Between Transferrin Receptor Concentration and Gestational Diabetes—Unadjusted Odds Ratio; Figure S10: Association Between Transferrin Receptor Concentration and Gestational Diabetes—Adjusted Odds Ratio; Figure S11: Hemoglobin Concentration (g/dL) Differences in GDM—Standardized Mean Difference—Weighted Mean Difference; Figure S12: Association Between Hemoglobin Concentration and GDM—Unadjusted Odds Rati; Figure S13: Association Between Hemoglobin Concentration and GDM—Adjusted Odds Ratio; Figure S14: Association Between Dietary Total Iron (No supplements) and GDM—Unadjusted Odds Ratio; Figure S15: Association Between Dietary Total Iron (No supplements) and GDM—Adjusted Odds Ratio; Figure S16: Association Between Dietary Non-heme Iron Intake and GDM—Unadjusted Odds Ratio; Figure S17: Association Between Dietary Non-heme Iron Intake and GDM—Unadjusted Odds Ratio; Figure S18: Association Between Dietary Heme Iron Intake (No supplements) and GDM—Unadjusted Odds Ratio; Figure S19: Association Between Dietary Heme Iron Intake (No supplements) and GDM—Adjusted Odds Ratio; Figure S20: Association Between Supplemental Iron Intake and GDM—Unadjusted Odds Ratio; Figure S21: Association Between Supplemental Iron Intake and GDM—Adjusted Odds Ratio; Figure S22: Sensitivity Analysis by Study Size; Figure S23: Sensitivity Analysis by Location; Figure S24: Sensitivity Analysis by Study Design; Figure S25: Leave One Out Analysis for Iron (SMD); Figure S26: Leave One Out Analysis for Ferritin (SMD); Figure S27: Leave One Out Analysis for Hemoglobin (SMD); Table S1: Study-specific Newcastle-Ottawa quality assessment.
