# Supplementary material for: Iron Status and Gestational Diabetes—A Meta-Analysis
Source: Nutrients. 2018 May 15;10(5):621. doi: 10.3390/nu10050621 (PMC5986501; doi:10.3390/nu10050621)

**Article Type:** Review article

**Title: Iron status and Gestational Diabetes – meta-analysis**

Yachana Kataria^1,2^, Yanxin Wu^1^ , Peter de Hemmer Horskjær^3^, Thomas Mandrup-Poulsen^3^, Christina Ellervik^1,2,3^

^1^ Department of Laboratory Medicine, Boston Children’s Hospital, Boston, USA

^2^ Harvard Medical School, Boston, USA

^3^ Faculty of Health and Medical Sciences, University of Copenhagen, Copenhagen, Denmark

**Corresponding author**:

Yachana Kataria, PhD, DABCC

300 Longwood Avenue, Boston, MA, 02215

Phone: 617-355-3159

Fax: 617-730-0383

[Yachana.Kataria@Childrens.harvard.edu](mailto:Yachana.Kataria@Childrens.harvard.edu)

**Conflict of Interest:**

There are no competing financial interests in relation to the work described.

## Abstract

**Objective**

A meta-analysis of the association of iron overload with gestational diabetes mellitus (GDM) may inform the health debate.

**Methods**

We performed a meta-analysis investigating the association of iron biomarkers and dietary iron exposure with GDM.

**Results**

We identified 33 eligible studies (N=44,110) published 2001-2017. The standardized mean differences (SMD) in women who had GDM compared to pregnant women without were 0.25 µg/dL (95% CI: 0.001-0.50) for iron, 1.54 ng/mL (0.56-2.53) for ferritin, 1.05% (0.02 to 2.08) for transferrin saturation, and 0.81 g/dL (0.40-1.22) for hemoglobin. Adjusted odds ratio for GDM were 1.58 (95% CI: 1.20-2.08) for ferritin, 1.30 (1.01-1.67) for hemoglobin, and 1.48 (1.29-1.69) for dietary heme intake. We did not find any differences in TIBC or transferrin concentration in women with and without GDM. We also did not find any association of increased transferrin receptor or increased intake of total dietary iron, non-heme iron or supplemental iron, with increased odds ratios for GDM. Considerable heterogeneity was present among the studies (0% - 99%) but no evidence of publication bias.

**Conclusions**

Accumulating evidence suggests that circulating and dietary iron biomarkers among pregnant women are associated with GDM, but results should be interpreted with caution due to high heterogeneity in analyses. Randomized trials investigating the benefits of iron reduction in women at high risk for GDM are warranted.

**Keywords:** iron, ferritin, transferrin, gestational diabetes, pregnancy

Abstract Word count: 217

## Introduction

Body iron is tightly regulated and dependent on nutritional needs and availability [1]. Adequate iron is critical for **β-**cell function and glucose homeostasis but excess iron beyond the need increases systemic oxidative stress [2]. Iron deficiency is common among pregnant women and remains a global public health concern. WHO recommends intake of 30 – 60 mg of elemental iron during pregnancy to prevent maternal iron deficiency anemia and to ensure adequate fetal iron stores [1]. However, health policy about the amount of iron supplementation during pregnancy varies between countries.

It is evident that pregnant women have an increased demand for iron due to physiological expansion of blood volume, needs of the fetus, and placental growth. Gestational diabetes mellitus (GDM) is associated with risks for the mom and the fetus. O’Sullivan et al. originally recommended the first screening and diagnostic criteria in the 1960s.[3] Since then, there has been debate in screening and diagnostic criteria for GDM. As such, various GDM diagnostic criteria have been adopted around the world by the governing agencies, and the criteria have been modified throughout the years.[3,4] Collectively, this results in a variable prevalence rate of GDM throughout the world. GDM is the most common metabolic disorder of pregnancy, and increasing incidence rates of GDM are thought to reflect the global rise in the incidence of type 2 diabetes mellitus (T2DM) related to energy intake in excess of energy expenditure.[5] Therefore, strategies addressing effective prevention, proper diagnosis and treatment are warranted. Fetal complications include obstetric complications related to macrosomia, neonatal hypoglycemia, perinatal mortality, congenital malformations, hyperbilirubinemia, polycythemia, hypocalcemia and respiratory distress syndrome.[6] The offspring are also at higher risk for glucose intolerance, diabetes and obesity in later years.[6] Maternal complications in women who develop GDM include hypertension, preeclampsia, cesarean delivery and increased risk of developing diabetes after pregnancy.[5,6]

It has been debated whether excess endogenous and exogenous (supplemental) iron is associated with GDM, but so far the studies have been inconsistent and heterogeneous with respect to measurement of iron exposure, ascertainment of GDM status, study design and population demographics [7-38]. There have been a few meta-analyses of observational studies published to date but they are not comprehensive and fail to provide conclusive evidence to determine if iron supplementation during pregnancy would pose any risk to development of GDM. Two previous randomized clinical trials in pregnant women failed to find any significant difference in the incidence of GDM in the iron supplement and placebo groups but these studies suffered from limitations in trial design, compliance, and ascertainment of exposure and outcome [19,39]. Most recently, Zhang et al. published a qualitative systematic review that critically examined the association between dietary iron intake, iron status and gestational diabetes, but the study did not provide quantitative estimates across the studies. Therefore, the aim of this study was to perform a quantitative, meta-analysis of the association of dietary iron intake, iron supplementation, and circulating iron biomarkers with GDM to inform the health debate.

## Methods

### *Study Strategy*

The study was conducted in accordance to Meta-analysis Of Observational Studies in Epidemiology (MOOSE) recommendations.

A systematic search of studies published before November 27^th^, 2017 was conducted utilizing a PubMed Search. The search was designed to capture records for articles describing the effect of iron intake and serum biomarkers of iron and GDM: ("iron"[MeSH Terms] OR "iron"[All Fields]) AND ("diabetes, gestational"[MeSH Terms] OR ("diabetes"[All Fields] AND "gestational"[All Fields]) OR "gestational diabetes"[All Fields] OR ("gestational"[All Fields] AND "diabetes"[All Fields])). Additional articles were captured by references and citations found in the original search and from the PubMed option “Similar Articles.”

*Exposure*

Exposure was defined as circulatory or dietary biomarkers of iron: hemoglobin (g/dL), ferritin (ng/mL), total iron binding capacity (TIBC) (µg/dL), transferrin (mg/dL), transferrin saturation (%), and transferrin receptor (mg/L). Dietary iron biomarkers included: total dietary iron with supplements, dietary iron without supplements, non-heme iron, heme iron, and supplemental iron.

#### Outcomes

A diagnosis of gestational diabetes status was based either on oral glucose tolerance test (OGTT), medical record, and/or self-report. More specifically, the OGTT diagnosis cut-off values utilized depend on which criteria were used by the study (i.e. WHO, ADA, and etc.). Specific criteria for each study are specified in Table 1.

#### Inclusion Criteria

Studies were selected if they reported an association of iron biomarkers and GDM.

The following criteria were required for eligibility: A) mean, standard deviation (SD), and N of the continuous exposures in pregnant women with vs. without GDM, or B) unadjusted and/or adjusted odds ratio and 95% CI for the binary outcomes in pregnant women with vs. without GDM, or C) raw N for the 2*2 tables to calculate the odds ratio and 95% CI for the binary outcomes in pregnant women with vs. without GDM.

#### Exclusion Criteria

We excluded abstracts, reviews, commentaries, studies with inadequate data, and other languages than English. Inadequate data was defined as data which was presented such that it was impossible to extract or calculate the necessary values for inclusion into a meta-analysis.

#### Study Selection Process

One author (YK) first examined study titles and abstracts. All studies identified as potentially relevant to the topic were eligible for a full text review. The study selection process is shown in Figure 1. List of selected studies can be found in Table 1. A total of 33 eligible studies were identified [7-38].

*Risk of bias and study quality assessment*

The quality of each study was evaluated and scored using the nine-star Newcastle-Ottawa Scale (NOS), a tool used for quality assessment of nonrandomized studies.[40] This was done independently by two authors (YK &YW). Studies were evaluated based on selection, comparability, exposure, and outcome, and scored by a maximum of nine points. Scores above five indicate moderate to high study quality. The NOS for cohort and case-control studies was retrieved from: [http://www.ohri.ca/programs/clinical_epidemiology/nosgen.pdf](https://urldefense.proofpoint.com/v2/url?u=http-3A__www.ohri.ca_programs_clinical-5Fepidemiology_nosgen.pdf&d=DwMFaQ&c=qS4goWBT7poplM69zy_3xhKwEW14JZMSdioCoppxeFU&r=AGqNrzpIResQVVFnZ7i0DkXbzKjRrZRu-xoGHierkO5VZVbHFh5P_OEGUqzLnYGW&m=2wH7xGWZ1eucWZjXYo0alLDsacLjtkFhJEySuPOJBwA&s=Uv58cy-jP7UJSMZd1wGP5mBjsYG8eM5Ujt2PueV2ja0&e=).

#### Data Extraction

Two authors (YK & YW) extracted information from the selected studies. The following variables were obtained from each study: author, size of study, year of publication, journal, name of study, assessment of GDM, GDM diagnosis criteria, assay type, the number of people with outcome/no outcome in exposed and non-exposed groups, odds ratio with 95% confidence interval (CI) or standard error (SE), mean and SD for continuous variables for exposed and non-exposed groups, and adjustment variables. Table 1 shows details of the selected studies and the data extracted from each study.

#### Data Synthesis and Meta-Analysis

Analysis was performed using STATA statistical software version 13.1. We combined summary estimates from all the studies in a meta-analysis.

We used the *metan* command to calculate random effects summary estimates in STATA. Comparing dietary and serum iron status to GDM vs. no GDM, we calculated weighted mean differences (WMD) and standardized mean differences (SMD) for continuous variables, and a pooled odds ratio for blood levels of ferritin, transferrin receptor, hemoglobin, and dietary iron intake. We meta-analyzed unadjusted and adjusted data separately. If studies presented odds ratios parsed by tertiles or quartiles of iron status, we calculated a within-study fixed effect odds ratio for binary GDM status (yes/no) before we meta-analyzed the odds ratios across studies. Statistical heterogeneity was assessed by the I^2^ statistic, which is a measure of between study variance, and the corresponding Cochran’s Q-statistic P-value (p(het)). To investigate validity and robustness of the meta-analysis of mean difference in iron, ferritin, and hemoglobin for which we had most studies, we explored heterogeneity in sensitivity analyses by stratifying on study size, continent, study design, and we performed leave-one-out analyses. Publication bias was measured by Begg’s test and Egger’s test and by visually assessing funnel plots.

## Results

After removing duplicates, 33 unique articles were identified (Figure 1). We included 33 studies in the meta-analysis on blood biomarkers of iron and dietary iron intake and its association with gestational diabetes. The New-Castle Ottawa qualitative assessment scale of bias revealed a score of 5-8 in case-control studies and 6-9 in cohort studies (Supplementary Table 1). Fifteen studies were from Middle East, six from Europe, six from Asia and six from US and one from Australia.

The studies were published from 2001 to 2017. In total, 44,110 pregnant women were included (age range 19 to 41 years).

### *Serum Iron*

The SMD and WMD of serum iron in women who had GDM compared to pregnant women without were 0.25 µg/dL (95% CI: 0.001 to 0.50; I^2^=83.4%, p(het)=3.10 x 10^-9^) and 11.31 µg/dL (95% CI: 0.87 to 21.75 I^2^=87.5%, p(het)=4.63 x 10^-13^), respectively (Figure 2 and Figures S1-2).

### *Serum Ferritin*

The SMD and WMD of serum ferritin in women who had GDM compared to pregnant women without were 1.54 ng/mL (95% CI: 0.56 to 2.53; I^2^=99.4.0%, p(het)= 0) and 11.33 mg/dL (95% CI: 6.68 to 15.97; I^2^=92.4%, p(het) =1.39 x 10^-25^), respectively (Figure 2, Figure 4, and Figure S3). Increased ferritin concentration was associated with GDM with an unadjusted odds ratio of 1.84 (95% CI: 1.51 to 2.23; I^2^=37.1%, p(het)=1.1 x 10^-1^) (Figure 2 and Figure S4) and an adjusted odds ratio of 1.58 (95% CI: 1.20 to 2.08; I^2^=0.0%, p(het)=3.83 x 10^-7^) (Figure 5 and Figure S4).

### *Total Iron Binding Capacity*

The SMD and WMD of total iron binding capacity (TIBC) in women who had GDM compared to pregnant women without were -0.47 ng/mL (95% CI: -1.07 to 0.14; I^2^=93.4%, p(het)= 8.0 x 10^-10^) and -28.14 mg/dL (95% CI: -63.86 to 7.57; I^2^=94.6%, p(het) =1.39 x 10^-25^), respectively (Figure 2 and Figures S5-6).

### *Transferrin Saturation and Transferrin Receptor*

The SMD and WMD of transferrin saturation in women who had GDM compared to pregnant women without were 1.05% (95% CI: 0.02 to 2.08; I^2^=93.6%, p(het)= 1.73 x 10^-7^) and 8.30% (95% CI: 1.38 to 15.22; I^2^=90.4%, p(het) =3.04 x 10^-5^), respectively (Figure 2 and Figures S7-8).

Increased transferrin receptor concentrations were associated with GDM with an unadjusted odds ratio of 1.32 (95% CI: 0.73 to 2.37; I^2^=99.8%, p(het)= 3.70 x 10^-206^) (Figure 3 and Figure S12) and an adjusted odds ratio of 1.18 (95% CI: 0.86 to 1.62; I^2^=77.8%, p(het)= 1.11 x 10^-2^) (Figure 3 and Figure S9-10).

### *Hemoglobin*

The SMD and WMD for hemoglobin levels in women who had GDM compared to pregnant women without were 0.81 g/dL (95% CI: 0.40 to 1.22; I^2^=95.9%, p(het)=3.69 x 10^-38^) and 0.44 g/dL (95% CI: 0.15 to 0.74; I^2^=93.9%, p(het)=2.33 x 10^-24^), respectively (Figure 2, Figure 6 and Figures S11). Increased hemoglobin concentration was associated with GDM with an unadjusted odds ratio of 1.34 (95% CI: 1.19 to 1.50; I^2^=22.5%, p(het)=2.71 x 10^-1^) (Figure 3 and Figure S12) and an adjusted odds ratio of 1.30 (95% CI: 1.01 to 1.67; I^2^=51.0%, p(het)=8.59 x 10^-2^) (Figure 3 and Figure S13).

### *Dietary Iron Biomarkers*

Total dietary iron not including supplements was not associated with GDM. The unadjusted OR was 0.96 (95% CI: 0.85 to 1.10; I^2^=78.6%, p(het)=9.28 x 10^-3^) (Figure 3 and Figure S14) and the adjusted OR was 1.08 (95% CI: 0.80 to 1.44; I^2^=64.9%, p(het)=3.58 x 10^-2^) (Figure 3 and Figure S15).

Dietary non-heme iron intake was not associated with GDM, with unadjusted OR of 0.80 (95% CI: 0.64 to 1.01; I^2^=81.8%, p(het)=9.00 x 10^-4^) and adjusted OR of 0.84 (95% CI: 0.64 to 1.11; I^2^=82.9%, p(het)=5.50 x 10^-4^) (Figure 3 and Figure S16-17).

Dietary heme iron intake was associated with GDM with an unadjusted OR of 1.53 (95% CI: 1.39 to 1.69; I^2^=0.0%, p(het)=5.89 x 10^-1^) and adjusted OR of 1.48 (95% CI: 1.29 to 1.69; I^2^=33.9%, p(het)=2.09 x 10^-1^) (Figure 3 and Figure S18-19).

Lastly, supplemental iron intake was not associated with GDM with an unadjusted OR of 1.20 (95% CI: 0.63 to 2.29; I^2^=93.7%, p(het)=2.26 x 10^-10^) and adjusted OR of 1.09(95% CI: 0.73 to 1.63; I^2^=82.7%, p(het)=3.00 x 10^-3^) (Figure 3 and Figure S20-21).

*Heterogeneity and Publication Bias and Sensitivity Analysis*

I^2^ heterogeneity varied from 0-99% but there was no publication bias present in the meta-analyses (Figure 2-3). Stratifying the meta-analysis by study size, showed that larger studies had smaller mean iron differences with narrow confidence intervals compared to smaller studies, but study size did not influence the mean differences observed for ferritin and hemoglobin concentrations (Figure S22). Stratifying the meta-analysis by geographical location revealed that studies from the Middle East and Asia had the larger and more variable mean differences in iron and hemoglobin levels (Figure S23), whereas mean differences in ferritin levels were higher among women of European descent. Stratification by study design revealed a variable pattern and heterogeneity (I^2^: 0-99%) amongst all studies for mean differences in iron, ferritin, and hemoglobin levels (Figure S24). Lastly, no gross changes were found in iron, ferritin, and hemoglobin mean differences compared to pooled sensitivity analysis conducted by leave-one-out analysis (Figure S25-27).

**Discussion**

There has been growing literature suggesting that elevated iron concentration is associated with the development of GDM. Understanding the relationship is imperative for designing future randomized control trials to determine if pregnant women can benefit from early intervention. We conducted a meta-analysis to determine if circulatory and dietary iron biomarkers are associated with development of gestational diabetes. The findings from this meta-analysis suggested that mean differences in circulating iron, ferritin, hemoglobin and transferrin saturation were higher in women with GDM compared to women without GDM, and increased ferritin, hemoglobin, and dietary heme intake were associated with increased odds ratios for GDM. We did not find any meaningful differences in TIBC and transferrin concentration in women with and without GDM. We also did not find any association of increased transferrin receptor or increased intake of total dietary iron, non-heme iron or supplemental iron, with increased odds ratio for GDM.

Ferritin concentrations were significantly and consistently associated with GDM across various observational studies and other meta-analyses [41-45]. Of note, the relationship persisted across different thresholds of high vs. low ferritin concentrations across different studies. It is also unclear what threshold of iron elevation is associated with GDM. Ferritin is a marker utilized to assess total body iron stores and it is also an acute phase reactant. The hormone hepcidin is the body’s main regulator of systemic iron homeostasis, and it is secreted in response to iron loading and inflammation.[46] Hepcidin has been associated with inflammatory states in humans.[46,47] The role of hepcidin in pregnancy is poorly understood, and only two studies have examined the association between GDM and hepcidin, both reporting increased hepcidin levels in GDM. [15,24] An increased maternal serum hepcidin level in GDM could potentially be due to increased body iron stores or inflammation. Interestingly, Rawal et al. found that the relationship persisted after adjusting for inflammation status (i.e. CRP levels). In a similar fashion, the association observed between ferritin and GDM could be reflective of underlying inflammation and not iron status. Moreover, markers of inflammation such as C-Reactive Protein (CRP) have also been associated with GDM [48]. To date, only a handful of studies simultaneously assessed the CRP and ferritin with GDM [10,15,38,49,50]. Chen et al. showed that individuals with higher serum ferritin and CRP levels had the greatest risk of GDM. This is consistent with Jiang et al. who has reported that high serum ferritin and CRP are independent risk factors for Type 2 diabetes [51]. Collectively this suggests that inflammation may mediate the development of GDM. However, in our analysis, other non-acute phase reactant markers such as serum iron and hemoglobin were also associated with GDM.

No single circulating iron biomarker alone is reflective of true iron status. Clinical utility of iron, ferritin, transferrin, transferrin saturation, soluble transferrin receptor are best when used in combination. However, the majority of the included studies failed to measure all iron biomarkers.

Serum iron measures the amount of circulating iron; our pooled estimates suggested that there was a positive and significant association between iron concentration and GDM. Animal model studies have confirmed that glucose tolerance is decreased by iron supplementation and is increased by iron depletion [52]. Whereas, transferrin, an iron transport protein, and transferrin saturation is readily calculated to serve as a sensitive indicator of functional iron deficiency. Our pooled estimates suggest that serum transferrin saturation is associated with GDM but not transferrin levels. Soluble transferrin receptor is increased in iron deficiency but unlike ferritin is not confounded by inflammation. All three studies published to date have found no significant association between transferrin receptor and GDM risk [13,15,38]. It is possible that no association was seen since transferrin receptor levels tend to rise only in the presence of a functional iron deficiency. Collectively, there were a limited number of studies examining these associations and thus results should be evaluated critically.

Hemoglobin concentration is affected by hemodilution during pregnancy, and a decreased concentration defines anemia; however, increased hemoglobin may be reflective of iron overload but may also reflect heavy smoking, high altitude, or a bone marrow disorder. In the meta-analysis, elevated hemoglobin concentration was associated with GDM.

Iron status of the body is regulated by dietary intake of the individual and also dependent on the type of dietary iron (i.e. heme vs. non-heme). Heme and non-heme iron are derived from different food sources and have different absorption mechanisms. Heme iron is derived largely from hemoglobin and myoglobin in meat, poultry, and fish. On the contrary plants, dairy products, and supplements are sources of non-heme iron. Dietary heme iron was positively and significantly associated with GDM but total dietary iron and non-heme iron were not associated with GDM. This finding could be attributable to the different absorption rates of heme and non-heme iron. Additionally the absorption of heme iron is less influence by other dietary factors compared to non-heme iron [53]. The negative and non-significant association of non-heme with GDM could be serving as a proxy for other dietary components that are reflective of a healthier diet but these findings are inconsistent. As such, fiber and polyunsaturated fats have been reported to be inversely associated with GDM risk [54,55].

In support of our findings, a case-control study showed that the iron overload C282Y allele frequency is higher in women with GDM than in healthy pregnant women, suggesting a genetic susceptibility to the development of GDM [56]. Our group has also previously shown that iron overload is associated with diabetes [57,58]. Higher levels of iron contribute to more reactive oxygen species and oxidative stress, ultimately leading to damage of the pancreatic beta cells and impairing insulin synthesis [59,60]. Reduction of the iron burden in type 2 diabetes animal models improve beta cell function [2]. It is also plausible that iron excess might interfere with glucose metabolism and trigger insulin resistance rather than impairing beta cells function. Animal models have shown that iron overload results in insulin resistance and hepatic glucose production [61]. This maybe mediated through the pancreatic beta-cells as it has a poor antioxidant capacity making them more susceptible to oxidative damage ultimately leading to impaired insulin synthesis.

Our meta-analysis is the most recent that comprehensively, critically, and quantitatively assesses the association between serum iron biomarkers and dietary biomarkers with gestational diabetes. Previous meta-analyses have either only qualitatively assessed the current data and/or are not comprehensive.[13,41,42,44,45] However, our study has limitations that should to be considered. Each meta-analysis had a variable number of studies included (3 to 12), which highlights the lack and need for additional studies assessing the association between iron status and GDM. Majority of the studies utilized either the ADA, WHO, or the Carpenter and Coustan OGTT diagnostic criteria. Assessing the studies by continent half of the studies (fifteen in total) were from Middle East, six from Europe, six from Asia five from US, and one from Australia. The GDM status was different in different regions of the world, and amount of glucose for OGTT varied between countries. These factors most likely contributed to the geographical heterogeneity which was observed in the meta-analyses. There were also studies with self-reported GDM which could introduce misclassification bias and potentially attenuate the observed results. Additionally, the selection of iron biomarkers to assess iron status varied among all the selected studies which could potentially introduce further biases in the observed results, and none of the studies used iron isotopes to measure iron absorption. Moreover, the heterogeneous selection of assays across studies may introduce bias, which may prevent us from observing a true association between iron status and GDM; however, use of the standardized mean difference largely reduces bias due to differences between assays. Additionally, almost all of the studies were observational in nature thus residual confounding can be expected. Recall bias may also be present in the questionnaire assessments of dietary iron intake. The presence of random error also attenuates the associations observed in this study and favors the null hypothesis.

The meta-analyses were based on observational studies, which are prone to confounding and reverse causation. Two previous randomized clinical trials (RCT) have been conducted investigating the effect of iron supplementation in healthy pregnant women and most with some degree of anemia, but did not find any association. [19,39] These studies suffered from limitations in trial design, compliance, and ascertainment of exposure and outcome. RCT studies would confirm findings seen across the literature and help determine if reducing iron status would prevent diabetes and/or improve glycemic control in patients with varying degrees of glucose intolerance; but to this date, no RCTs have been conducted in women with first time GDM or in women with a second pregnancy with a previous history of GDM investigating the effects of iron supplementation on the mother and the fetus. These studies are needed to identify if certain high-risk groups could benefit from iron reduction or simply no iron supplement during pregnancy. Furthermore, concerns regarding iron supplementation in women with adequate levels ought to be investigated.

In conclusion, the meta-analysis suggested that mean differences in circulating iron, ferritin, hemoglobin and transferrin saturation were higher in women with GDM compared to women without GDM, and increased ferritin, hemoglobin, and dietary heme intake were associated with increased odds ratios for GDM. Results should be interpreted with caution as the pooled estimates had high heterogeneity potentially due to geographical locations, GDM assessment, selection of iron biomarkers and assays. The lack of association does not necessarily support the null hypothesis as some analyses included only a few studies and thus have wider confidence intervals. There is a clear need for larger and systematically conducted randomized controlled trials utilizing all the available iron biomarkers to elucidate the potential benefit and risk of iron repletion among replete pregnant women and in pregnant women at high risk of developing GDM.

**Conflict of Interest:**

There are no competing financial interests in relation to the work described.

**Funding:**

The authors have no support or funding to report.

**References**

1. Benoist, B.d.; McLean, E.; Egll, I.; Cogswell, M. Worldwide prevalence of anaemia 1993-2005: Who global database on anaemia. *Worldwide prevalence of anaemia 1993-2005: WHO global database on anaemia.* **2008**.

2. Hansen, J.B.; Moen, I.W.; Mandrup-Poulsen, T. Iron: The hard player in diabetes pathophysiology. *Acta Physiol (Oxf)* **2014**, *210*, 717-732.

3. O'SULLIVAN, J.B.; Mahan, C.M. Criteria for the oral glucose tolerance test in pregnancy. *Diabetes* **1964**, *13*, 278.

4. Organization, W.H. Diagnostic criteria and classification of hyperglycaemia first detected in pregnancy. **2013**.

5. Chiefari, E.; Arcidiacono, B.; Foti, D.; Brunetti, A. Gestational diabetes mellitus: An updated overview. *Journal of endocrinological investigation* **2017**, *40*, 899-909.

6. Setji, T.L.; Brown, A.J.; Feinglos, M.N. Gestational diabetes mellitus. *Clinical diabetes* **2005**, *23*, 17-24.

7. Mari-Sanchis, A.; Diaz-Jurado, G.; Basterra-Gortari, F.J.; de la Fuente-Arrillaga, C.; Martinez-Gonzalez, M.A.; Bes-Rastrollo, M. Association between pre-pregnancy consumption of meat, iron intake, and the risk of gestational diabetes: The sun project. *Eur J Nutr* **2017**.

8. Behboudi-Gandevani, S.; Safary, K.; Moghaddam-Banaem, L.; Lamyian, M.; Goshtasebi, A.; Alian-Moghaddam, N. The relationship between maternal serum iron and zinc levels and their nutritional intakes in early pregnancy with gestational diabetes. *Biol Trace Elem Res* **2013**, *154*, 7-13.

9. Bowers, K.; Yeung, E.; Williams, M.A.; Qi, L.; Tobias, D.K.; Hu, F.B.; Zhang, C. A prospective study of prepregnancy dietary iron intake and risk for gestational diabetes mellitus. *Diabetes Care* **2011**, *34*, 1557-1563.

10. Chen, X.; Scholl, T.O.; Stein, T.P. Association of elevated serum ferritin levels and the risk of gestational diabetes mellitus in pregnant women: The camden study. *Diabetes Care* **2006**, *29*, 1077-1082.

11. Darling, A.M.; Mitchell, A.A.; Werler, M.M. Preconceptional iron intake and gestational diabetes mellitus. *Int J Environ Res Public Health* **2016**, *13*.

12. Helin, A.; Kinnunen, T.I.; Raitanen, J.; Ahonen, S.; Virtanen, S.M.; Luoto, R. Iron intake, haemoglobin and risk of gestational diabetes: A prospective cohort study. *BMJ Open* **2012**, *2*.

13. Khambalia, A.Z.; Aimone, A.; Nagubandi, P.; Roberts, C.L.; McElduff, A.; Morris, J.M.; Powell, K.L.; Tasevski, V.; Nassar, N. High maternal iron status, dietary iron intake and iron supplement use in pregnancy and risk of gestational diabetes mellitus: A prospective study and systematic review. *Diabet Med* **2016**, *33*, 1211-1221.

14. Qiu, C.; Zhang, C.; Gelaye, B.; Enquobahrie, D.A.; Frederick, I.O.; Williams, M.A. Gestational diabetes mellitus in relation to maternal dietary heme iron and nonheme iron intake. *Diabetes Care* **2011**, *34*, 1564-1569.

15. Rawal, S.; Hinkle, S.N.; Bao, W.; Zhu, Y.; Grewal, J.; Albert, P.S.; Weir, N.L.; Tsai, M.Y.; Zhang, C. A longitudinal study of iron status during pregnancy and the risk of gestational diabetes: Findings from a prospective, multiracial cohort. *Diabetologia* **2017**, *60*, 249-257.

16. Soheilykhah, S.; Mojibian, M.; Jannati Moghadam, M. Serum ferritin concentration in early pregnancy and risk of subsequent development of gestational diabetes: A prospective study. *Int J Reprod Biomed (Yazd)* **2017**, *15*, 155-160.

17. Soubasi, V.; Petridou, S.; Sarafidis, K.; Tsantali, C.; Diamanti, E.; Buonocore, G.; Drossou-Agakidou, V. Association of increased maternal ferritin levels with gestational diabetes and intra-uterine growth retardation. *Diabetes Metab* **2010**, *36*, 58-63.

18. Tarim, E.; Kilicdag, E.; Bagis, T.; Ergin, T. High maternal hemoglobin and ferritin values as risk factors for gestational diabetes. *International Journal of Gynecology & Obstetrics* **2004**, *84*, 259-261.

19. Chan, K.K.; Chan, B.C.; Lam, K.F.; Tam, S.; Lao, T.T. Iron supplement in pregnancy and development of gestational diabetes--a randomised placebo-controlled trial. *BJOG* **2009**, *116*, 789-797; discussion 797-788.

20. Afkhami-Ardekani, M.; Rashidi, M. Iron status in women with and without gestational diabetes mellitus. *J Diabetes Complications* **2009**, *23*, 194-198.

21. Al-Saleh, E.; Nandakumaran, M.; Al-Rashdan, I.; Al-Harmi, J.; Al-Shammari, M. Maternal-foetal status of copper, iron, molybdenum, selenium and zinc in obese gestational diabetic pregnancies. *Acta Diabetol* **2007**, *44*, 106-113.

22. Al-Saleh, E.; Nandakumaran, M.; Al-Shammari, M.; Al-Harouny, A. Maternal-fetal status of copper, iron, molybdenum, selenium and zinc in patients with gestational diabetes. *J Matern Fetal Neonatal Med* **2004**, *16*, 15-21.

23. Amiri, F.N.; Basirat, Z.; Omidvar, S.; Sharbatdaran, M.; Tilaki, K.H.; Pouramir, M. Comparison of the serum iron, ferritin levels and total iron-binding capacity between pregnant women with and without gestational diabetes. *J Nat Sci Biol Med* **2013**, *4*, 302-305.

24. Derbent, A.U.; Simavli, S.A.; Kaygusuz, I.; Gumus, II; Yilmaz, S.; Yildirim, M.; Uysal, S. Serum hepcidin is associated with parameters of glucose metabolism in women with gestational diabetes mellitus. *J Matern Fetal Neonatal Med* **2013**, *26*, 1112-1115.

25. Kaygusuz, I.; Gumus, II; Yilmaz, S.; Simavli, S.; Uysal, S.; Derbent, A.U.; Gozdemir, E.; Kafali, H. Serum levels of visfatin and possible interaction with iron parameters in gestational diabetes mellitus. *Gynecol Obstet Invest* **2013**, *75*, 203-209.

26. Javadian, P.; Alimohamadi, S.; Gharedaghi, M.H.; Hantoushzadeh, S. Gestational diabetes mellitus and iron supplement; effects on pregnancy outcome. *Acta Med Iran* **2014**, *52*, 385-389.

27. Ozyer, S.; Engin-Ustun, Y.; Uzunlar, O.; Katar, C.; Danisman, N. Inflammation and glycemic tolerance status in pregnancy: The role of maternal adiposity. *Gynecol Obstet Invest* **2014**, *78*, 53-58.

28. Sharifi, F.; Ziaee, A.; Feizi, A.; Mousavinasab, N.; Anjomshoaa, A.; Mokhtari, P. Serum ferritin concentration in gestational diabetes mellitus and risk of subsequent development of early postpartum diabetes mellitus. *Diabetes Metab Syndr Obes* **2010**, *3*, 413-419.

29. Wang, Y.; Tan, M.; Huang, Z.; Sheng, L.; Ge, Y.; Zhang, H.; Jiang, M.; Zhang, G. Elemental contents in serum of pregnant women with gestational diabetes mellitus. *Biol Trace Elem Res* **2002**, *88*, 113-118.

30. Akhlaghi, F.; Bagheri, S.M.; Rajabi, O. A comparative study of relationship between micronutrients and gestational diabetes. *ISRN Obstet Gynecol* **2012**, *2012*, 470419.

31. Pan, J.; Zhang, F.; Zhang, L.; Bao, Y.; Tao, M.; Jia, W. Influence of insulin sensitivity and secretion on glycated albumin and hemoglobin a1c in pregnant women with gestational diabetes mellitus. *Int J Gynaecol Obstet* **2013**, *121*, 252-256.

32. Lao, T.T.; Chan, L.Y.; Tam, K.F.; Ho, L.F. Maternal hemoglobin and risk of gestational diabetes mellitus in chinese women. *Obstet Gynecol* **2002**, *99*, 807-812.

33. Lao, T.T.; Chan, P.L.; Tam, K.F. Gestational diabetes mellitus in the last trimester - a feature of maternal iron excess? *Diabet Med* **2001**, *18*, 218-223.

34. Bo, S.; Menato, G.; Villois, P.; Gambino, R.; Cassader, M.; Cotrino, I.; Cavallo-Perin, P. Iron supplementation and gestational diabetes in midpregnancy. *Am J Obstet Gynecol* **2009**, *201*, 158 e151-156.

35. Palma, S.; Perez-Iglesias, R.; Prieto, D.; Pardo, R.; Llorca, J.; Delgado-Rodriguez, M. Iron but not folic acid supplementation reduces the risk of low birthweight in pregnant women without anaemia: A case-control study. *J Epidemiol Community Health* **2008**, *62*, 120-124.

36. Gungor, E.S.; Danisman, N.; Mollamahmutoglu, L. Maternal serum ferritin and hemoglobin values in patients with gestational diabetes mellitus. *Saudi medical journal* **2007**, *28*, 478-480.

37. Tan, P.; Chai, J.; Ling, L.; Omar, S. Maternal hemoglobin level and red cell indices as predictors of gestational diabetes in a multi-ethnic asian population. *Clinical and experimental obstetrics & gynecology* **2011**, *38*, 150-154.

38. Bowers, K.A.; Olsen, S.F.; Bao, W.; Halldorsson, T.I.; Strom, M.; Zhang, C. Plasma concentrations of ferritin in early pregnancy are associated with risk of gestational diabetes mellitus in women in the danish national birth cohort. *J Nutr* **2016**, *146*, 1756-1761.

39. Kinnunen, T.I.; Luoto, R.; Helin, A.; Hemminki, E. Supplemental iron intake and the risk of glucose intolerance in pregnancy: Re-analysis of a randomised controlled trial in finland. *Matern Child Nutr* **2016**, *12*, 74-84.

40. Stang, A. Critical evaluation of the newcastle-ottawa scale for the assessment of the quality of nonrandomized studies in meta-analyses. *European journal of epidemiology* **2010**, *25*, 603-605.

41. Fernandez-Cao, J.C.; Aranda, N.; Ribot, B.; Tous, M.; Arija, V. Elevated iron status and risk of gestational diabetes mellitus: A systematic review and meta-analysis. *Matern Child Nutr* **2017**, *13*.

42. Fu, S.; Li, F.; Zhou, J.; Liu, Z. The relationship between body iron status, iron intake and gestational diabetes: A systematic review and meta-analysis. *Medicine (Baltimore)* **2016**, *95*, e2383.

43. Han, S.; Middleton, P.; Shepherd, E.; Van Ryswyk, E.; Crowther, C.A. Different types of dietary advice for women with gestational diabetes mellitus. *Cochrane Database Syst Rev* **2017**, *2*, CD009275.

44. Zhao, L.; Lian, J.; Tian, J.; Shen, Y.; Ping, Z.; Fang, X.; Min, J.; Wang, F. Dietary intake of heme iron and body iron status are associated with the risk of gestational diabetes mellitus: A systematic review and meta-analysis. *Asia Pac J Clin Nutr* **2017**, *26*, 1092-1106.

45. Zhang, C.; Rawal, S. Dietary iron intake, iron status, and gestational diabetes. *Am J Clin Nutr* **2017**, *106*, 1672S-1680S.

46. Ganz, T. Hepcidin and iron regulation, 10 years later. *Blood* **2011**, *117*, 4425-4433.

47. Nemeth, E.; Rivera, S.; Gabayan, V.; Keller, C.; Taudorf, S.; Pedersen, B.K.; Ganz, T. Il-6 mediates hypoferremia of inflammation by inducing the synthesis of the iron regulatory hormone hepcidin. *The Journal of clinical investigation* **2004**, *113*, 1271-1276.

48. Li, X.; Lu, X. Study on correlation between c-reactive protein and gestational diabetes mellitus. *Journal of Nanjing Medical University* **2007**, *21*, 382-385.

49. Zein, S.; Rachidi, S.; Awada, S.; Osman, M.; Al-Hajje, A.; Shami, N.; Sharara, I.; Cheikh-Ali, K.; Salameh, P.; Hininger-Favier, I. High iron level in early pregnancy increased glucose intolerance. *J Trace Elem Med Biol* **2015**, *30*, 220-225.

50. Khambalia, A.Z.; Collins, C.E.; Roberts, C.L.; Morris, J.M.; Powell, K.L.; Tasevski, V.; Nassar, N. Iron deficiency in early pregnancy using serum ferritin and soluble transferrin receptor concentrations are associated with pregnancy and birth outcomes. *Eur J Clin Nutr* **2016**, *70*, 358-363.

51. Jiang, R.; Manson, J.E.; Meigs, J.B.; Ma, J.; Rifai, N.; Hu, F.B. Body iron stores in relation to risk of type 2 diabetes in apparently healthy women. *JAMA* **2004**, *291*, 711-717.

52. Dongiovanni, P.; Valenti, L.; Ludovica Fracanzani, A.; Gatti, S.; Cairo, G.; Fargion, S. Iron depletion by deferoxamine up-regulates glucose uptake and insulin signaling in hepatoma cells and in rat liver. *Am J Pathol* **2008**, *172*, 738-747.

53. Bothwell, T.H. Overview and mechanisms of iron regulation. *Nutrition reviews* **1995**, *53*, 237-245.

54. Wang, Y.; Storlien, L.H.; Jenkins, A.B.; Tapsell, L.C.; Jin, Y.; Pan, J.F.; Shao, Y.F.; Calvert, G.D.; Moses, R.G.; Shi, H.L.*, et al.* Dietary variables and glucose tolerance in pregnancy. *Diabetes Care* **2000**, *23*, 460-464.

55. Zhang, C.; Liu, S.; Solomon, C.G.; Hu, F.B. Dietary fiber intake, dietary glycemic load, and the risk for gestational diabetes mellitus. *Diabetes Care* **2006**, *29*, 2223-2230.

56. Sivitskaya, L.; Danilenko, N.; Zabarouskaya, Z.; Davydenko, O. Hfe gene mutation associated with the severity of gestational diabetes mellitus in belarusian women. *Age (years)* **2013**, *28*, 5.7.

57. Bonfils, L.; Ellervik, C.; Friedrich, N.; Linneberg, A.; Sandholt, C.H.; Jørgensen, M.E.; Jørgensen, T.; Hansen, T.; Pedersen, O.; Allin, K.H. Fasting serum levels of ferritin are associated with impaired pancreatic beta cell function and decreased insulin sensitivity: A population-based study. *Diabetologia* **2015**, *58*, 523-533.

58. Ellervik, C.; Mandrup-Poulsen, T.; Andersen, H.U.; Tybjærg-Hansen, A.; Frandsen, M.; Birgens, H.; Nordestgaard, B.G. Elevated transferrin saturation and risk of diabetes: Three population-based studies. *Diabetes care* **2011**, *34*, 2256-2258.

59. Backe, M.B.; Moen, I.W.; Ellervik, C.; Hansen, J.B.; Mandrup-Poulsen, T. Iron regulation of pancreatic beta-cell functions and oxidative stress. *Annual review of nutrition* **2016**, *36*, 241-273.

60. Cejvanovic, V.; Kjær, L.K.; Bergholdt, H.K.M.; Torp-Pedersen, A.; Henriksen, T.; Weimann, A.; Ellervik, C.; Poulsen, H.E. Iron induced rna-oxidation in the general population and in mouse tissue. *Free Radical Biology and Medicine* **2018**, *115*, 127-135.

61. Leong, W.-I.; Lönnerdal, B. Iron nutrition. In *Iron physiology and pathophysiology in humans*, Springer: 2012; pp 81-99.

## Table & Figure Legend

**Table 1**: Study Characteristics

**Figure 1**: Selection of Studies

**Figure 2**: Blood and Dietary Iron Biomarkers and Gestational Diabetes Status (Mean Difference)

**Figure 3:** Differences in iron and Gestational Diabetes Status (Odds Ratio)

**Figure 4:** Ferritin Concentration (ng/mL) Differences in GDM – Standardized Mean Differences

**Figure 5:** Adjusted Association Between Ferritin Concentration and GDM – Adjusted Odds Ratio

**Figure 6:** Hemoglobin Concentration (g/dL) Differences in GDM – Standardized Mean Difference

**Supplementary Tables and Figures**

1. Table S1 Study-specific Newcastle Ottawa quality assessment
2. Figure S1 - Iron Concentration (µg/dL) Differences in GDM – Standardized Mean Differences
3. Figure S2 – Iron Concentration (µg/dL) Differences in GDM – Weighted Mean Difference
4. Figure S3 - Ferritin Concentration (ng/mL) Differences in GDM – Weighted Mean Difference
5. Figure S4 - Association Between Ferritin Concentration and GDM – Unadjusted Odds Ratio
6. Figure S5 - TIBC Concentration (µg/dL) Differences in GDM –Standardized Mean Differences
7. Figure S6 - TIBC Concentration (µg/dL) Differences in GDM – Weighted Mean Differences
8. Figure S7 - Transferrin Saturation (%) Differences in GDM – Standardized Mean Differences
9. Figure S8 - Transferrin Saturation (%) Differences in GDM – Weighted Mean Differences
10. Figure S9 - Association Between Transferrin Receptor Concentration and Gestational Diabetes – Unadjusted Odds Ratio
11. Figure S10 - Association Between Transferrin Receptor Concentration and Gestational Diabetes – Adjusted Odds Ratio
12. Figure S11 - Hemoglobin Concentration (g/dL) Differences in GDM – Standardized Mean Difference – Weighted Mean Difference
13. Figure S12 - Association Between Hemoglobin Concentration and GDM – Unadjusted Odds Ratio
14. Figure S13 - Association Between Hemoglobin Concentration and GDM – Adjusted Odds Ratio
15. Figure S14 - Association Between Dietary Total Iron (No supplements) and GDM – Unadjusted Odds Ratio
16. Figure S15 - Association Between Dietary Total Iron (No supplements) and GDM – Adjusted Odds Ratio
17. Figure S16 - Association Between Dietary Non-heme Iron Intake and GDM – Unadjusted Odds Ratio
18. Figure S17 - Association Between Dietary Non-heme Iron Intake and GDM – Adjusted Odds Ratio
19. Figure S18 - Association Between Dietary Heme Iron Intake (No supplements) and GDM – Unadjusted Odds Ratio
20. Figure S19 - Association Between Dietary Heme Iron Intake (No supplements) and GDM – Adjusted Odds Ratio
21. Figure S20 - Association Between Supplemental Iron Intake and GDM – Unadjusted Odds Ratio
22. Figure S21 - Association Between Supplemental Iron Intake and GDM – Adjusted Odds Ratio
23. Figure S22 – Sensitivity Analysis by Study Size
24. Figure S23 – Sensitivity Analysis by Location
25. Figure S24 – Sensitivity Analysis by Study Design
26. Figure S25 – Leave One Out Analysis for Iron (SMD)
27. Figure S26 – Leave One Out Analysis for Ferritin (SMD)
28. Figure S27 – Leave One Out Analysis for Hemoglobin (SMD)


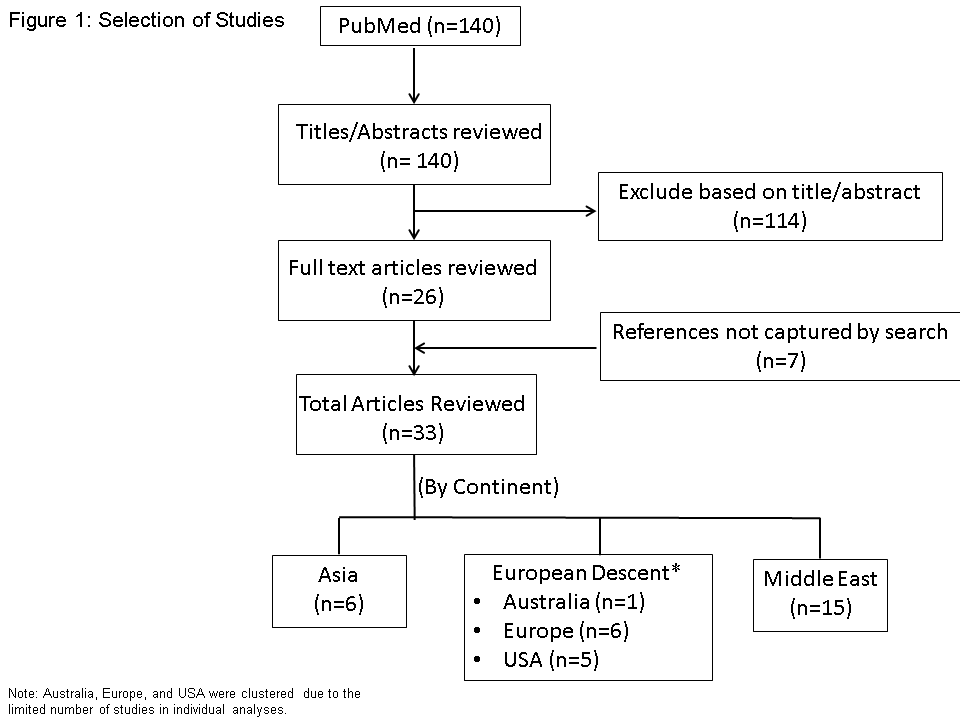

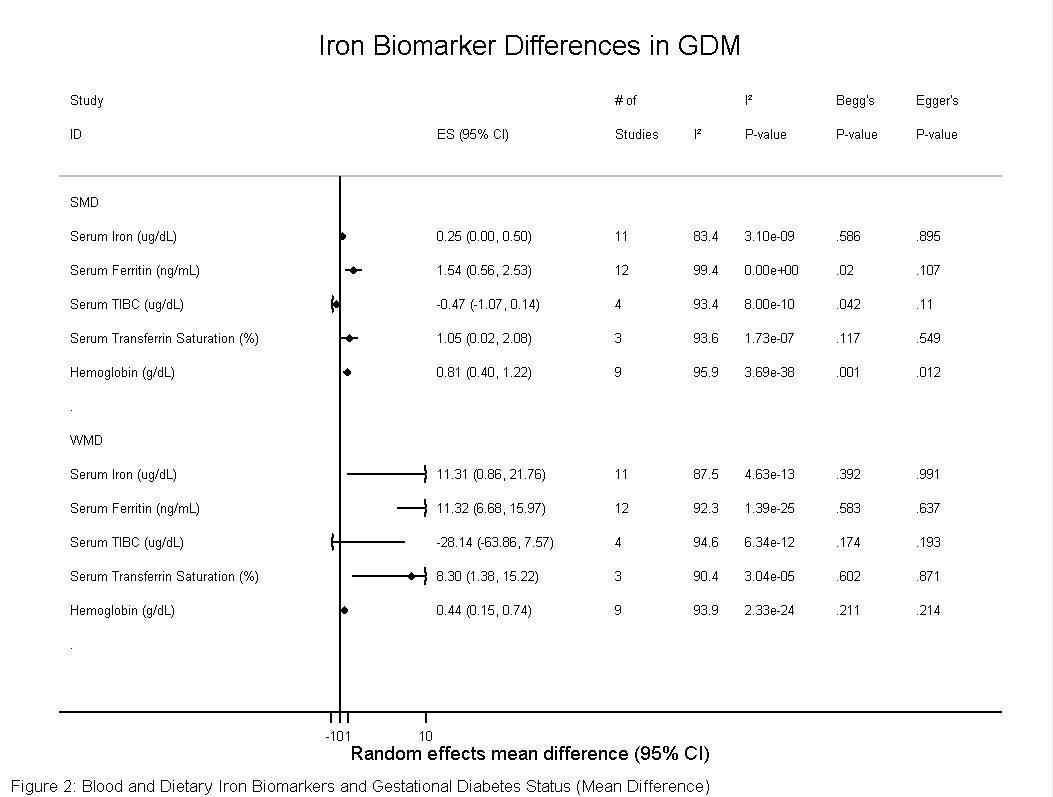

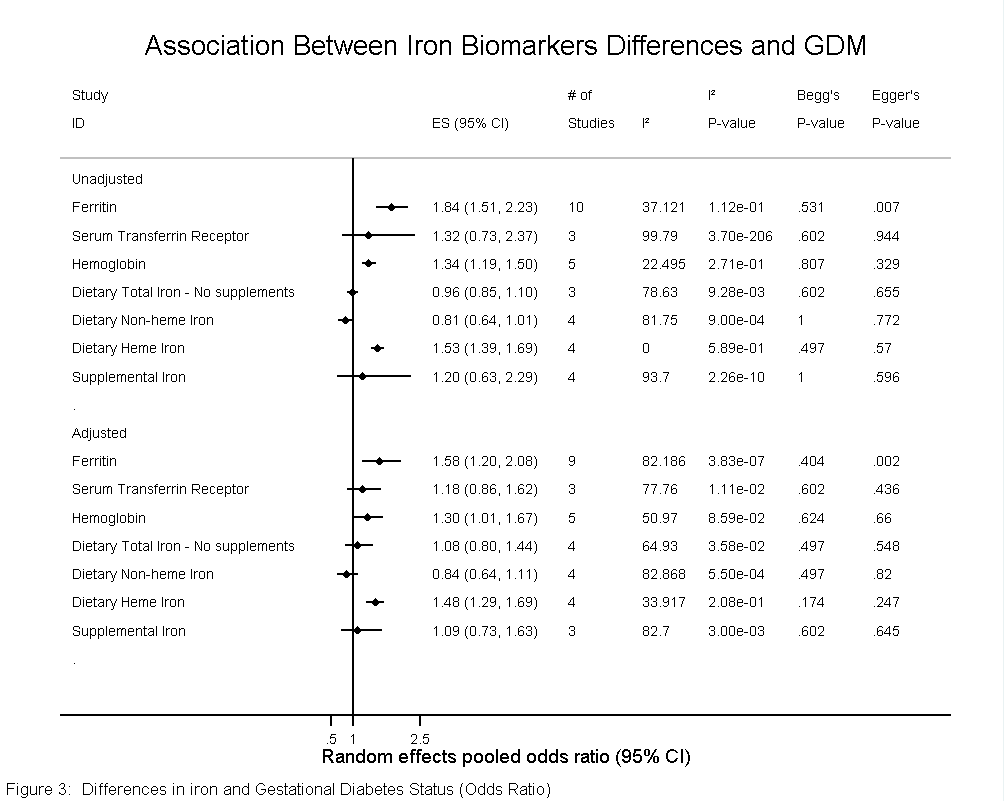


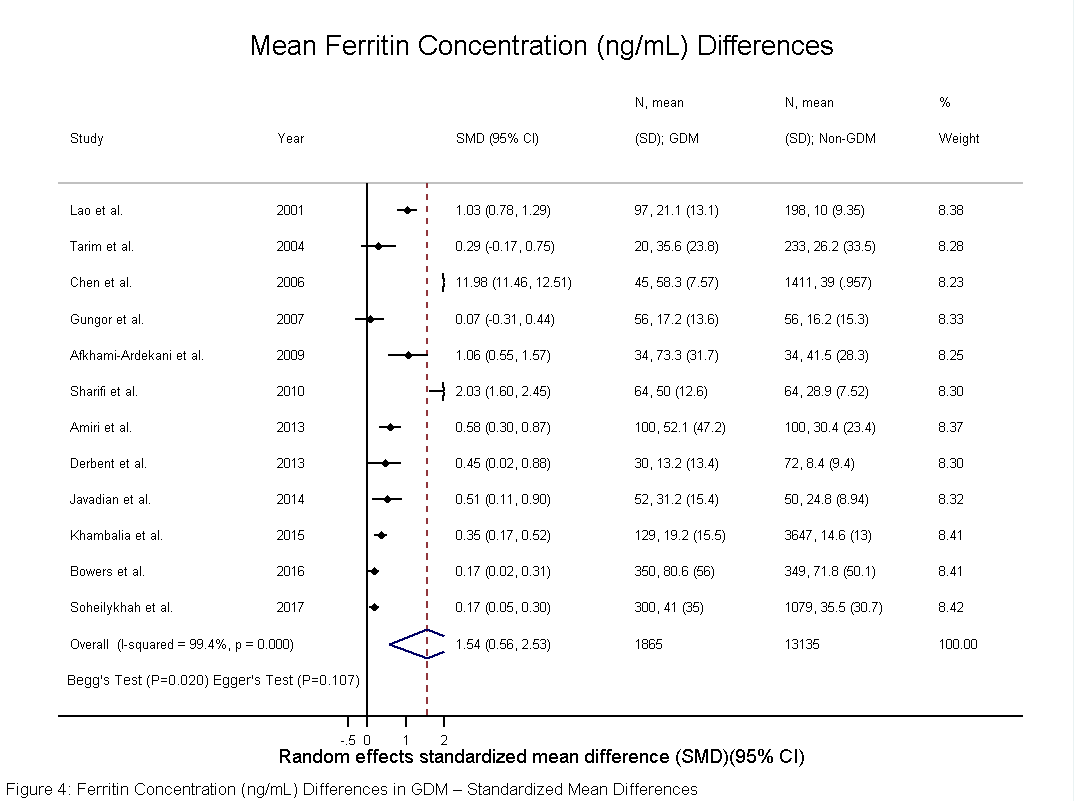

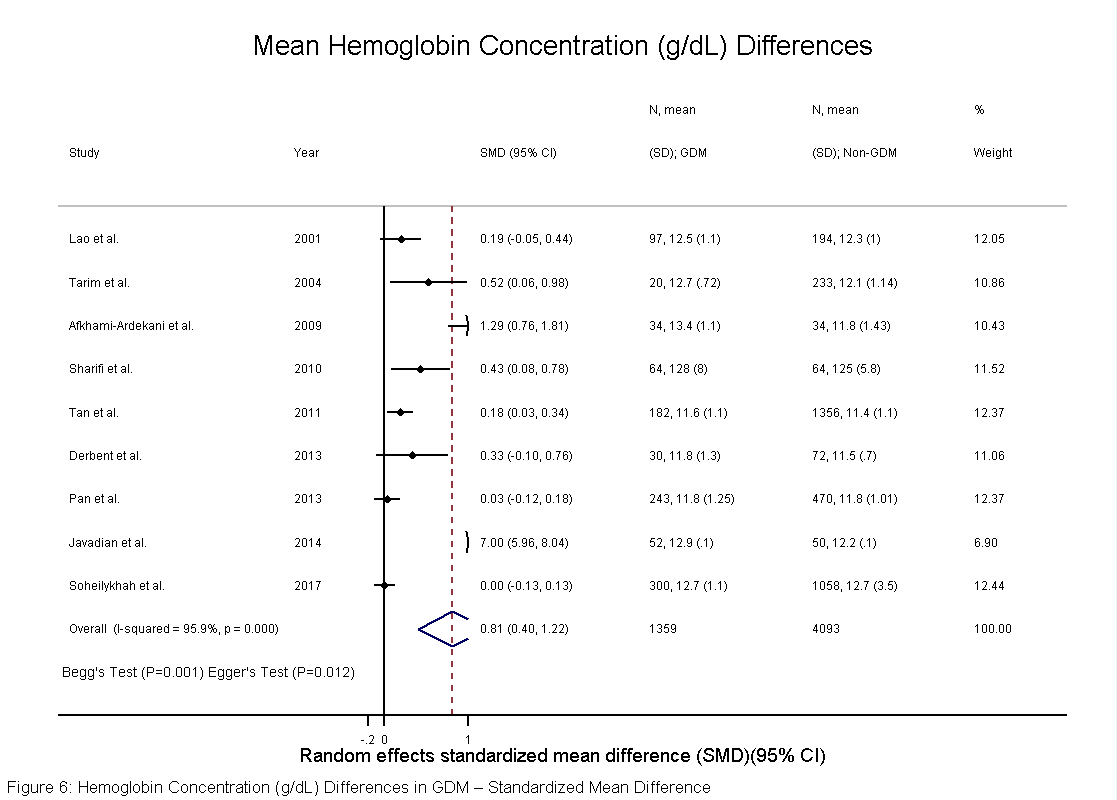

Supplement: Supplementary file 1 [file nutrients-10-00621-s001.zip › Iron_Manuscript_4302019_TrackChanges.docx]
