# Supplementary material for: Iron Status and Gestational Diabetes—A Meta-Analysis
Source: Nutrients. 2018 May 15;10(5):621. doi: 10.3390/nu10050621 (PMC5986501; doi:10.3390/nu10050621)
Supplement: Supplementary file 1 [file nutrients-10-00621-s001.zip › SupplmentalFigures_3292018.docx]

Figure S1. Iron Concentration (µg/dL) Differences in GDM – Standardized Mean Differences

**Figure S2. Iron Concentration (µg/dL) Differences in GDM – Weighted Mean Difference**

Figure S3. Ferritin Concentration (ng/mL) Differences in GDM – Weighted Mean Difference

Figure S4. Association Between Ferritin Concentration and GDM – Unadjusted Odds Ratio

Figure S5. TIBC Concentration (µg/dL) Differences in GDM –Standardized Mean Differences

**Figure S6. TIBC Concentration (µg/dL) Differences in GDM – Weighted Mean Differences**

Figure S7. Transferrin Saturation (%) Differences in GDM – Standardized Mean Differences

Figure S8. Transferrin Saturation (%) Differences in GDM – Weighted Mean Differences

Figure S9. Association Between Transferrin Receptor Concentration and Gestational Diabetes – Unadjusted Odds Ratio

**Figure S10. Association Between Transferrin Receptor Concentration and Gestational Diabetes – Adjusted Odds Ratio**

Figure S11. Hemoglobin Concentration (g/dL) Differences in GDM – Standardized Mean Difference – Weighted Mean Difference

**Figure S12. Association Between Hemoglobin Concentration and GDM – Unadjusted Odds Ratio**

**Figure S13. Association Between Hemoglobin Concentration and GDM – Adjusted Odds Ratio**

**Figure S14. Association Between Dietary Total Iron (No supplements) and GDM – Unadjusted Odds Ratio**

**Figure S15. Association Between Dietary Total Iron (No supplements) and GDM – Adjusted Odds Ratio**

Figure S16. Association Between Dietary Non-heme Iron Intake and GDM – Unadjusted Odds Ratio

Figure S17. Association Between Dietary Non-heme Iron Intake and GDM – Unadjusted Odds Ratio

Figure 18.

**Figure S18. Association Between Dietary Heme Iron Intake (No supplements) and GDM – Unadjusted Odds Ratio**

**Figure S19. Association Between Dietary Heme Iron Intake (No supplements) and GDM – Adjusted Odds Ratio**

**Figure S20. Association Between Supplemental Iron Intake and GDM – Unadjusted Odds Ratio**

Figure S21. Association Between Supplemental Iron Intake and GDM – Adjusted Odds Ratio

**Figure S22. Sensitivity Analysis by Study Size**

**Figure S23. Sensitivity Analysis by Location**

Figure S24. Sensitivity Analysis by Study Design

Figure S25. Leave One Out Analysis for Iron (SMD)

Figure S26. Leave One Out Analysis for Ferritin (SMD)

Figure S27. Leave One Out Analysis for Hemoglobin (SMD)
